# Assessment of Frequency and Causes of Medication Errors in Pediatrics and Emergency Wards of Teaching Hospitals Affiliated to Tehran University of Medical Sciences (24 Hospitals)

**DOI:** 10.25122/jml-2018-0046

**Published:** 2018

**Authors:** Fatemeh Izadpanah, Shekoufeh Nikfar, Freshteh Bakhshi Imcheh, Mina Amini, Marzieh Zargaran

**Affiliations:** 1. Food and Drug Administration of Iran, Tehran, Iran; 2. Iran Council for Review and Formulation of Drugs, Tehran, Iran; 3. Mazandaran University of Medical Sciences, Sari, Iran; 4. Pharmacoeconomics and Pharmaceutical Administration, Tehran University of Medical Sciences. Tehran, Iran

**Keywords:** Medication errors, causes of errors, pediatrics wards, emergency ward, hospital

## Abstract

**Introduction and Objective:** Medical errors and adverse events are among the major causes of avoidable deaths and costs incurred on health systems all over the world. Medical errors are among the main challenges threatening the safety of patients in all countries and one of the most common types of medical errors is medication errors. This study aimed to determine the frequency, type, and causes of medication errors in the emergency and pediatric wards of hospitals affiliated to Tehran University of Medical Sciences in 2017.

**Materials and Methods:** This study was a cross-sectional descriptive study which was conducted on 423 nurses working in teaching hospitals affiliated to Tehran University of Medical Sciences in 2017. The subjects were selected using the stratified sampling method. A total of 49 teaching hospitals in Tehran are affiliated to Tehran University of Medical Sciences and they are divided into two groups of general and specialized hospitals. Of all, 10 general hospitals and 14 specialized hospitals were randomly selected. The required data was collected using a three-part questionnaire. Using the SPSS software (version 18), the collected data was analyzed by means of ANOVA, Pearson Correlation Coefficient, and t-test and the results were reported as frequency, percentage, mean, and standard deviation.

**Results:** According to the results of this study, the mean total number of medication errors that occurred within one month in the pediatric and emergency wards was roughly 41.9 cases, as stated by the nurses. The mean number of medication errors was higher in men than in women. Also, the two variables of gender and the type of shift work were related to medication errors; specifically, it was higher first in the evening and night shifts and then in the morning and evening shifts, respectively. Also, the number was higher in night shifts than in the morning shifts. The most common types of medication errors were: administration of the drugs at the wrong time, using a wrong technique of administration, wrong dosage, forgetting the dosage of the drug, administrating additional doses, administrating the drug to a wrong patient, and following the oral orders of physicians. On the other hand, the most common causes of medication errors in clinical wards were the following: illegible physician orders, shortage of manpower and high workload, incomplete physician orders, the use of lookalike and sound-alike drugs, absence of pharmacist/pharmaceutical expert in the ward, lack of dosage forms appropriate for children, and lack of adequate training regarding drug therapy.

**Discussion and Conclusion:** Considering the results of this study, it is necessary to reduce the workload and working hours of nurses, increase medical staff’s awareness of the significance of medication errors, revise the existing techniques of drug prescription, and update the indices of human resource in hospitals. It is also necessary to correct the process of naming and selecting the dosage forms of drugs by the industry.

## Introduction

Medical errors and adverse events are among the major causes of avoidable deaths and costs incurred on health systems all over the world and medical errors are among the main challenges threatening the safety of patients. One of the most common types of medical errors is inappropriate medication use. In 2000, the American National Academy of Sciences issued a publication titled “To err is human” and reported that every year, about 98,000 patients in the country die due to medical errors, which was indicative of the low quality of health care provided for the patients.

Therefore, extensive planning was made in order to increase the quality of care provided and increase patient safety. Currently, unwanted medical errors affect one out of every 10 patients around the world; as a result, the World Health Organization has recognized the problem as an endemic problem [1].

About 10–16% of hospitalized patients experience medical errors or adverse events. Half of the cases are manageable and preventable. In developing countries, no clear statistics about the incidence of medical errors are reported. However, the lack of clear statistical data about medication errors does not imply that there is no medication error [2]. The primary and expectable outcome of such errors is the lengthened duration of hospitalization and increased expenses, and occasionally severe harm and even death [3].

Meanwhile, although clear statistics about medical errors in Iran are not available, the experts predict that due to some structural weaknesses in the national health system, the rate of medical errors must be very high. According to the Ministry of Health and Medical Education, billions of dollars are spent every year to provide care services for hospitalized patients who have become a victim to medical errors and thus have been faced with complications and prolonged stay in hospitals [4].

These errors could directly inflict damage upon the patient and increase health care costs and could indirectly harm the healthcare personnel on professional and personal levels and reduce their self-esteem and performance [5, 6, 13].

About one third to one-half of adverse side effects of drugs is due to medication errors. Such errors can occur at any stage of the medication process in which medical technicians & nurses are involved and more prone to cause medication errors [7, 8, 12]. According to the available statistical data, more than 50 medication orders are administered by a nurse in a single shift [9, 13] and it accounts for about one-third of the nurses’ work time [10, 11]. For Instance, based on the results of a study in a hospital, the prevalence of medication errors among nurses was 79% [4, 14].

On average, nurses spend 40% of their time in the hospital to give medicines to the patients and based on the previous studies, the rate of serious damage to patients owed to medication errors are vastly oscillating between 1–2% and 51.8% [4, 15].

Five important rules should be observed by nurses while administering drugs for patients: correct patient, right medication, the right way to administer medication, right dosage, and the right time. The mentioned items must be observed carefully when administering any type of drug [4, 16]. According to most studies, medication errors in children are more common than in adults with a greater risk of death [13, 17, 18]. Medication errors in children can occur at any step of the treatment process and it may occur because of an individual or systematic error [13, 19]. Ordering, dispensing, and administering medications, and monitoring the drug therapy process in children are more complex and challenging [13, 20].

For example, to determine the proper dosage of all medications it is necessary to determine the child’s weight and consequently, more operations are usually needed to calculate the proper drug regimen for children. In addition, as the solution of the drugs for children should be diluted, there is a higher probability of error in the preparation of drugs. On the other hand, children lack the communication skills to avoid potential errors in medication which may cause side effects. Moreover, children’s bodies, especially babies’, are physiologically weak and have a reduced capability of compensating for medication errors [13, 21]. The errors can occur in every step of the medication process including prescribing, physicians transcribing the drugs, reading the prescriptions, dispensing, administering, or controlling the drugs [4, 22, 23].

Other variables which may be involved in medication errors are the following: similarity in shape and packaging of drugs, inappropriate space for dispensing the drugs, inadequate number of employees, staff fatigue and carelessness, low pharmaceutical knowledge of nurse [4, 25, 26].

Several strategies have been proposed by various studies to prevent and reduce medication errors [4, 23, 27, 28] among which we may note the following: staff training, communicating information regarding new drugs, availability of pharmacology books in clinical wards, and holding pharmaceutical conferences. Moreover, the full-time presence of pharmacologists in the hospital can increase the nurses’ easy access to pharmaceutical information. Determining the prevalence and type of medication errors could help to propose some solutions or make some decisions regarding appropriate and safe medication administration. The above-mentioned measures could be taken in order to prevent medication errors and thus increase the quality of care and safety for patients [4, 38].

The aim of this study was to assess the status of medication errors in teaching hospitals and to propose strategies for reducing these mistakes in Iran’s health system.

## Materials and Methods

This study was a cross-sectional descriptive study which was conducted on 423 nurses working in teaching hospitals affiliated to Tehran University of Medical Sciences in 2017. The subjects were selected using the stratified sampling method. A total of 49 teaching hospitals in Tehran are affiliated to Tehran University of Medical Sciences and they are divided into two groups: general and specialized hospitals. Of all, 10 general hospitals and 14 specialized hospitals were randomly selected, and afterward, the smallest acceptable sample size was determined. The required data was collected using a three-part questionnaire. The first part included demographic information regarding education, experience and so forth. The second part collected data about a variety of medication errors of nurses that occurred during the past month. The third section was used to collect data concerning the causes of errors. In the errors section, nurses were asked to write the number of each type of error which had occurred during the given period; the number of errors could range from zero to any other number. The third part of the questionnaire included questions with a five-point Likert scale rating from 1 to 5 (1=the least important, and 5=the most important). At the end of the questionnaire, an open question asked the subjects to propose some methods to reduce medication errors. This questionnaire was completely filled in by nurses.

To verify the content validity of the questionnaire, we collected the views of three faculty members from the Pharmacy department and six other faculty members from other departments. The reliability of the questionnaire was verified using intraclass correlation index and internal consistency was verified by Cronbach’s alpha test. The Cronbach’s alpha was 0.826 and minimum and maximum intraclass correlation, respectively, were 0.452 and 0.856 (acceptable range).

Using SPSS software (version 18) the collected data was analyzed by means of ANOVA, Pearson correlation coefficient, and t-test and the results were reported as frequency, percentage, mean, and standard deviation.

## Results

The mean age of the nurses was 32.48 years, ranging from 23 to 49 years. The mean work experience was 7.5 years, ranging from one year to 32 years. Of all, 32.38% of the subjects were single and 67.61% were married. Moreover, 48.46% of the subjects had rotating work shifts and 51.54% had a fixed work shift. The majority of cases (69.97%) had been formally employed or had a contract. Of all, 31.2% of the subjects had passed the pharmaceutical training courses. Considering the education level, 80.85% had a bachelor’s degree and the remaining had a master’s degree. The majority of nurses (70.02%) only worked in one hospital. According to the results of this study, the mean total number of medication errors occurred at the fault of nurses during a month in pediatrics and emergency wards was about 41.9 cases. The two variables of gender and the type of shift work were significantly related to medication errors and the mean number of medication errors was higher in men than in women. As mentioned, it was higher first in the evening and night shifts and then in the morning and evening shifts, respectively; furthermore, the mean number of medication errors was higher in night shifts than in the morning shifts. However, there was no statistically significant difference between the numbers of medication errors in the morning shift and rotating shifts. There was no statistically significant relationship between the number of medication errors and age, work experience, marital status, type of employment, history of attending training courses, and working in one or more hospitals (Table 1).

Considering the type and the mean number of medication errors, the most common types found in a month were: administration of drugs at the wrong time, using a wrong technique administration, wrong dosage, forgetting the dosage indications, administrating additional doses, administrating the drug to a wrong patient, and following the verbal orders of physicians (Table 2).

Other common causes of medication errors in clinical wards were: illegible physician orders, shortage of manpower and high workload, incomplete physician orders, the use of look-alike and sound-alike drugs, absence of a pharmacist/pharmaceutical expert in the ward, lack of dosage forms appropriate for children, lack of adequate training about drug therapy, illegible drug charts and wrong calculations. But also fatigue caused by workload, working long and continuous shifts, inadequate wages, lack of motivation and family problems (Table 3).

## Discussion and Conclusion

According to the results of this study, the mean total number of medication errors occurred at the nurses’ fault during a month in pediatrics and emergency wards was approximately 41.9 cases, which is higher in comparison with the results of other studies. Therefore, more attention must be paid to this problem as error rates are increasing. According to the results of Hajibabaei et al.’s study in 2011, the mean total number of medication errors of each nurse within three months was 19.5 [29]. According to the results of another study by Panjwini et al. in Sanandaj, the percentage of medication error was 16.7% in 2001 [30]. Based on Mrayyan et al.’s study in 2005, the mean number of medication errors for each nurse was 2.2 cases per every three months [31]. Stratton et al. stated in 2004 that the rate of medication errors was 14.8 cases per 1,000 patients [17]. Finally, as reported by Lisby et al. in 2005, the rate of the incidence of medication errors in Denmark was 43% [32].

**Table 1:** Relationship between frequency distribution of medication errors and occupational and demographic characteristics of the nurses participating in the study

Variable		Number of nurses (%)	Mean number of errors
Gender	Female	384 (88.8)	32.2 ± 3.2
	Male	39 (11.2)	51.6 ± 5.7
Marital status	Married	286 (67.61)	42.8 ± 3.1
	Single	137 (32.38)	40.7 ± 3.7
Working in more than one clinical center	Yes	126 (29.78)	45.3 ± 4.4
	No	297 (70.02)	36.2 ± 2.7
Type of shift work	Morning and evening	14 (3.31)	52.7 ± 5.8
	Evening and night	28 (6.62)	68.9 ± 7.1
	Morning	106 (25.06)	24.3 ± 2.8
	Night	51 (12.06)	34.1 ± 3.1
	Rotating	205 (48.46)	27.2 ± 2.7
Type of employment	Official	124 (29.31)	38.2 ± 3.8
	Contract	172 (40.66)	45.9 ± 4.7
	Mission	71 (16.78)	42.4 ± 3.7
	Specific contract	56 (13.24)	34.9 ± 2.8
Attending training courses	Yes	132 (31.20)	40.7 ± 3.9
	No	291 (68.80)	39.9 ± 3.7
Education level	Bachelor	342 (80.85)	47.5 ± 4.1
	Master	81 (19.15)	34.9 ± 3.8
Work experience	≥ 5 Years	138 (32.62)	46.9 ± 4.1
	5> years	285 (67.38)	32.6 ± 3.6

**Table 2:** Frequency distribution of different types of medication errors

No.	Type of error	Percentage
1	administration of the drugs at the wrong time	30.6
2	wrong technique	21.1
3	wrong dosage	17.7
4	missing a medication dose	16.3
5	administrating additional doses of drugs	8.1
6	administrating the drug to a wrong patient	3.5
7	following the verbal orders of physicians	1.5
8	others	1.2

**Table 3:** Frequency distribution of causes of medication errors

No.	Cause of medication error	Percentage
1	illegible physician orders	27.5
2	shortage of manpower and high workload	21.3
3	incomplete physician orders	16.2
4	the use of look-alike and sound-alike drugs	14.3
5	absence of pharmacist/pharmaceutical expert in the ward	11.1
6	lack of dosage forms appropriate for children	4.1
7	lack of adequate training for drug therapy	2.7
8	illegible drug chart	1.3
9	wrong calculations	1.1
10	others	0.4

There was no significant relationship between the incidence of medication errors and employment status. This finding is similar to the results of a study by Zahmatkeshan et al., where there was no significant relationship between medication errors and work experience; this finding is consistent with the results of studies by Yousefi et al. (2017) [34], Zahmatkeshan et al. (2001) [33], and Hajibabaei et al. (2011) [29]. However, the studies by Sheu et al. (2009) [35] and Ito et al. (2003) [36] showed that with increasing work experience the incidence of medication errors decreases.

This study showed that with increasing the working hours, the number of medication errors also increased; this finding is in line with the results of studies by Lerner et al. (2008) [37], Madadi et al. (2015) [4], Shield et al. (2008) [38], and Taheri et al. (2012) [39]. In addition, the number of medication errors which occurred at night was higher; this might be due to the conflict between the working hours at night and the circadian rhythm which could lead to sleep disturbances and decreased concentration and attention and cause increased irritability and fatigue. This finding is consistent with the results of a study by Bagheri and Valizadeh (2006) [40]. Hajibabaei et al.’s study (2011) also showed an increase in errors during the night shift and attributed these errors to sleep deprivation, physiological changes, disorders in heart rhythm, and reduced concentration that ultimately led to the poor performance and alertness of the personnel working in night shifts [29].

According to the results of Seki and Yamasaki’s study (2006) [34, 41], there was no statistically significant difference between the number of medication errors that occurred in different work shifts. However, the studies by Rosen Robert et al. (2004) [42], Tisdale et al. (1986) [43], Booker et al. (1995) [44], Sozani et al. (2007) [45], and Panjwini et al. (2006) [30] showed that the highest number of medication errors occurred during the morning shift.

According to the results of this study, the incidence of medication errors was associated with the gender of nurses; accordingly, medication errors were more prevalent in male nurses than in females. This finding is consistent with the results of studies by Hajibabaei et al., Maryyan et al. (2007) [31], Yousefi et al. (2017) [34], and Kohestani et al. (2010) [33].

In this study, the most common medication errors were the following: administrating drugs at the wrong time (earlier or later than the scheduled time), administration with a wrong technique (wrong route of administration), incorrect intravenous infusion rate, mixing two or more drugs in the micro-set regardless of drug interactions, forgetting a dose or giving extra doses, following the verbal orders of physicians, administering several oral medications at once, using a wrong dosage of drugs, administering a wrong drug for the patient, not diluting the drug, and not observing the time interval required between drug administration and food consumption. Our finding is in line with the results of Wilkins and Shields et al.’s study (2008) [38] which reported that 54% of errors were related to the wrong time of drug administration. Similarly, Raja Lope et al. (2009) [46] conducted a study in NICU and showed that the maximum number of errors was related to the administration of the drug at the wrong time. According to a study by Prot et al. (2003) [47] the most common medication errors were the following: wrong time of drug administration (36%), wrong medication technique (19%), wrong dose (15%), administering drugs without a physician prescription (10%), and using an inappropriate form of drug (8%). In Yousefi et al.’s study (2017) [34] the most common medication errors were: administering drugs at the wrong time, using drugs without a physician order, following verbal orders, mixing two or more drugs in the micro-set regardless of possible interactions, and eliminating a dose of the drug; these findings are rather similar to the results of our study. Gholamnejad and Nickpima’s study [48] indicates that the most common medication errors are the use of a wrong dosage of medication (27%), eliminating a dose of the drug (22%), and wrong time of drug administration (18%). In another study conducted by Cheraphi et al. (2011) [49], the most common medication errors were incorrect infusion rate (44.68%) and wrong dose (23.4%). In a study by Tang et al. (2007) [24] the most prevalent medication errors were the use of a wrong dose (36.1%) and the use of a wrong drug (26.4%). Alijanzadeh et al. (2015) [50] reported incorrect intravenous infusion rate as the most common medication error occurred in clinical wards, which is consistent with the results of a study by Kaushal et al. (2001) [21] that was conducted in a specialized pediatrics clinical center. Ebrahimpur et al.’s study showed that the highest rate of medication errors was related to the administration of drugs to the wrong patient [51].

Regarding the most important causes of medication errors in clinical wards noted in Table 2, our results were confirmed by Pournamdar et al. [52]. These findings are similar to the results of Yousefi et al.’s study (2013) that reported the following as the most common causes of medication errors: high workload of nurses, shortage of nurses in proportion to the number of patients in each ward, fatigue due to extra tasks, long and continuous shifts, similar drug packaging and drug name, lack of time, and wards being filled to capacity [34].

The results of Sozani et al.’s study (2007) showed that fatigue caused by additional tasks, lack of balance between the number of nurses and patients, mental and health problems, illegible physician orders written in patients’ files, and shortage of time play a major role in the incidence of medication errors [45]; these findings are consistent with the results of our study.

According to Tang et al.’s study (2007), an increased workload is the second cause of medication errors which is in line with the results of our study [24]. Nickpima and Gholamnejad (2010) showed that the main causes of medication errors included excessive workload, inadequate number of personnel, physical and emotional fatigue, and continuous and excessive working hours, while other authors also had results that were consistent with the outcomes of this study [48, 49, 57].

According to our results, in order to deal with the factors that play a major role in the incidence of medication errors, it is necessary to take corrective measures. For instance, it is necessary to reduce the workload and working hours of nurses because fatigue and loss of concentration can increase pitfalls. It is also necessary to raise medical staff’s awareness of the importance of medication errors and the need for reforming the methods of transcribing medical prescriptions because this issue needs to be addressed sooner rather than later and lack of planning for reforms in this area will lead to increased rate of errors. Moreover, it is of great importance to have pharmaceutical experts in every ward because they can monitor and follow up all the tasks relating to drugs; on the other hand, the presence of pharmaceutical experts can reduce the workload of nurses. In general, upgrading the human resource index in hospitals can have an important role in reducing medication errors. The pharmaceutical industry can modify the naming process and determine the type of drugs, thus it can largely prevent medication errors caused by look-alike and sound-alike drugs. Holding practical training courses on drug recognition and calculation can greatly help to improve the process of drug prescription and administration. Hence, it is suggested to direct the policies and programs made by healthcare providers toward achieving such goals.

### Study limitation

We met some limitations in this study of which the most important cases are listed below:

Fear of losing the confidence and respect of patients and colleagues, fear of disciplinary actions, doubt regarding receiving a low rating and being blamed, incertitude of significant changes and corrective processes after error reporting. In this manner, nurses’ workload affected their correct responses.

## Acknowledgment

We want to express our gratitude to all the people working in teaching hospitals affiliated to Tehran University of Medical Sciences who actively participated in the implementation of this study. It is hoped that all stakeholders will benefit from this study through revising processes that will lead to reducing the number of medication errors.

## Conflict of Interest

The authors confirm that there are no conflicts of interest.

## References

[1] Cherry B, Jacob S. Contemporary Nursing. *Mosby*.

[2] Rahimi S, Seyyed-rasouli A (2004). Nurses’ Drug Precautions Awareness. *Iranian Journal of Nursing*.

[3] Rouhi Boroujeni H, Deris F, Izadpanah F (2017). *IIOABJ*.

[4] Madadi Z, Jaafaripooyan E (2015). Nursing Medication Errors, Causes and Solutions. *J of Hospital*.

[5] Anderson DJ, Webster CS (2001). A System Approach to the Reduction of Medication Errors on the Hospital Ward. *J. of Advanced Nursing*.

[6] Mayo AM, Duncan D (2004). Nurses’ Perception of Medication Error: What We Need for Patient Safety. *Journal of Nursing Care Quality*.

[7] William DJP (2007). Medication Error. *Journal of Royal College of Physicians of Edinburgh*.

[8] Fontan JE, Maneglier V, Nguyen VX, Loirat C, brion F (2003). Medication Errors in Hospitals, Computerized Unit Dose Drug Dispensing Systems Versus Ward Stock Distribution System. *Pharmacy World Science*.

[9] Morris S (1991). Who’s to Blame?. *Nursing*.

[10] Wakefield Bj (2005). Development and Validation of the Medication Administration Error Reporting Survey. *Advances in Patient Safety*.

[11] Pepper GA (1995). Error in Drug Administration by Nurses. *American Journal of Health System Pharmacy*.

[12] Bergqvist M. (2012). Medication Errors by Nurses in Sweden, Classification and Contributing Factors. Open Access Scientific Reports.

[13] Oladi Ghadikalaee R, Ravaghi H, Hesam S (2015). Study of Nurses’ Perceptions on Medication Errors in Pediatric Hospitals in Tehran. *Payavarde Salamat*.

[14] Mirzaei M, Khatony A, R. F. (2013). Prevalence and Type of Nursing Medication Errors and Barriers to Reporting by Nurses in a Teaching Hospital of Kermanshah. *Journal of Nursing and Midwifery School*.

[15] Jember Abebaw, Hailu Mignote, Messele Anteneh, Demeke Tesfaye, Hassen Mohammed (2018). *BMC Nursing*.

[16] Phillips J, Beam S, Brinker A (2001). Retrospective Analysis of Mortalities Associated with Medication Errors. *Am J Health Syst Pharm.*.

[17] Stratton KM, Blegen MA, Pepper G, Vaughn T. (2004). Report of Medication Errors by Pediatric Nurses. *Journal of Pediatric Nursing*.

[18] Gladstone J. (1995). Drug Administration Error. *Journal of Advanced Nursing*.

[19] Antonow JA, Smith AB, Silver MP (2000). Medication Error Reporting. *Journal of Nursing Care Quality*.

[20] Taheri E, Nourian M, Rasouli M, Kavousi A (2013). The Study of Type & Amount of Medication Errors in Neonatal Intensive Care Units and Neonatal Units. *Iranian Journal of Critical Care Nursing*.

[21] Koushal R, Botes DW, Londrigon C (2001). Medication Errors and Adverse Drug Events in Pediatric Inpatients. *Journal of American Medical Association*.

[22] Lehmann CU, Conner KG, Cox JM (2004). Preventing Provider Errors: Online Total Parenteral Nutrition Calculator Pediatrics.

[23] Hughes RG, Ortiz E (2005). Medication Errors: Why They Happen, and How They Can Be Prevented. *Journal of Infusion Nursing*.

[24] Tang FI, Sheu SJ, Yu S, Wei IL, Chen CH (2007). Nurses Relate the Contributing Factors Involved in Medication Errors. *Journal of Clinical Nursing*.

[25] Koohestani H, Baqcheghi N (2008). Survey of Medication Errors at Nursing Students in Cardiac Care Unit. *Journal of Forensic Medicine*.

[26] Zahmatkeshan N, Bagherzadeh R, Mirzaie K (2010). An Observational Study to Evaluate the Medication Errors by Nursing Staff Working in Bushehr Medical Centers During One Year Interval (2006–2007). *Iranian South Medical Journal*.

[27] Ghassemi F, Valizadeh F, Momennasab M (2005). Nurses’ Opinions and Knowledge About Medication Error and Preventive Solutions in University Hospitals Of Khorramabad. *Journal of Lorestan University of Medical Sciences*.

[28] Bates DW, Teich JM, Lee J, Seger D, Kuperman GJ, Ma’Luf N (1999). The Impact of Computerized Physician Order Entry on Medication Error Prevention. *Journal of the American Medical Informatics Association*.

[29] Hajibabaiee F, Jolaee S, Payravi H, Bagani H (2012). The Relationship of Medication Errors Among Nurses with Some Organizational and Demographic Characteristics. *Iranian Journal of Nursing Research*.

[30] Penjvini S (2006). Investigation of the Rate and Type of Medication Errors of Nurses in Sanandaj Hospitals. *Iranian Journal of Nursing Research*.

[31] Mrayyan M T, Shishani K, Al-Faouri I (2007). Rate, Causes and Reporting of Medication Error Errors in Jordan: Nurses’ Perspectives. *Journal of Nursing Management*.

[32] Lisby M, Nielsen LP, Mainz J (2005). Errors in The Medication Process: Frequency, Type and Potential Clinical Consequences. *International Journal of Quality Healthcare*.

[33] Baghcheghi N, Koohestani H R (2010). The Comments of Nursing Educators About Reasons and Reduction Strategies of Medication Errors in Nursing Students in Arak University of Medical Sciences, 2008. *Arak Medical University Journal (AMUJ)*.

[34] Yousefi M S, Abed Saeedi Zh, Maleki M, Sarbakhsh P (2014). Frequency and Causes of Medication Errors of Nurses in Different Shift Works in Educational Hospitals Affiliated to Shahid Beheshti University of Medical Sciences. *J. of Nursing & Midwifery Shahid Beheshti University of Medical Sciences*.

[35] Sheu S J, Wei IL, Chen CH, Yu S, Tang FI (2009). Using Snowball Sampling Method with Nurses to Understand Medication Administration Errors. *Journal of Clinical Nursing*.

[36] Ito H, YamaZumi S (2003). Common Types of Medication Errors on Long-Term Psychiatric Care Units. International Journal for Quality in Health Care.

[37] Lerner RB, Carvalho Md, Vieira AA, Lopes JM, Moreira ME (2008). Medication Errors in Neonatal Care Unit. *Journal de Pediatria*.

[38] Wilkins K, Shields M (2008). Correlates of Medication Errors Hospital. Health Reports.

[39] Taheri E (2012). Study of Type and Amount of Medication Errors and Relevant Factors in Neonatal Intensive Care Units. Hospitals Affiliated to Shahid Beheshti University of Medical Sciences 2011 (Dissertation).

[40] Baghery M, Valizadeh Nagemeh N (2006). Night Work and its Consequences on Nurses’ Health. *Journal of Gorgan Bouyeh Faculty of Nursing & Midwifery*.

[41] Seki Y, Yamazaki Y (2006). Effects of Working Conditions on Intravenous Medication Errors in a Japanese Hospital. *Journal of Nursing Management*.

[42] Rosen Robert K (2004). Medication Errors: a 21st Century Perspective. *Health Care Research and Improvement*.

[43] Tisdale J E (1986). Justifying a Pediatric Critical-Care Satellite Pharmacy by Medication Error Reporting. *American Journal of Hospital Pharmacy*.

[44] Booker G M, Roseman M (1995). Workload and Environmental Factors in Hospital Medication Errors. *Nursing Research*.

[45] Soozani A, Bagheri H, Poorheydari M (2007). Nurses’ Perspective on Causes of Medication Errors in Shahrood. *Journal of Knowledge and Health*.

[46] Raja Lope RJ, Boo NY, Rohana J, Cheah FC (2009). A Quality Assurance Study on the Administration of Medication by Nurses in Neonatal Intensive Care Unit. *Sangapor Medical Journal*.

[47] Prot S, Fontan JE, Alberti C (2005). Drug Administration Errors and their Determinants in Pediatric Inpatients. *International Journal for Quality of Health Care*.

[48] Nikpeyma N, Gholamnejad H (2009). Reasons for Medication Errors in Nurses’ Views. *Journal of Shahid Beheshti Faculty of Nursing and Midwifery*.

[49] Cheraghi M, Nikbakht A, MohammadNejad E, Salari A, Ehsani R. (2011). Incidence of Nursing Medication Errors in ICU. *Journal of Mazandarn University of Medical Sciences*.

[50] Alijanzadeh M., Mohebifar R., Azadmanesh Y., Faraji M. (2015). The Frequency of Medication Errors and Factors Influencing the Lack of Reporting Medication Errors in Nursing at Teaching Hospital of Qazvin University of Medical Sciences (2012). *Journal of Health*.

[51] Ebrahimpour F, Shakrokhi A, A. G. (2014). Patient Safety and Nurses’ Medication Administration Errors. *Journal of Forensic Medicine*.

[52] Pournamdar Zahra, Zare Sadegh (2016). *Biology & Medicine*.

